# Integration of single-cell and bulk RNA sequencing to establish a prognostic signature based on tumor-associated macrophages in colorectal cancer

**DOI:** 10.1186/s12876-023-03035-4

**Published:** 2023-11-10

**Authors:** Hua Li, Lujuan Pan, Junyu Guo, JianLe Lao, Mingwei Wei, Fuda Huang

**Affiliations:** 1 Department of Anorectal Surgery, The Affiliated Hospital of Youjiang Medical University for Nationalities, Baise, Guangxi Province China; 2 Gastroenterology Department, The Affiliated Hospital of Youjiang Medical University for Nationalities, Baise, Guangxi Province China; 3 Department of Cardiothoracic Surgery, The Affiliated Hospital of Youjiang Medical University for Nationalities, Baise, Guangxi Province China

**Keywords:** Tumor-associated macrophage, Prognostic signature, Immunotherapy, Tumor microenvironment, Colorectal cancer

## Abstract

Several studies have shown significant involvement of tumor-associated macrophages (TAMs) in the tumor microenvironment and cancer progression. However, no data on reliable TAM-related biomarkers are available for predicting the prognosis of patients with colorectal cancer (CRC). We analyzed the clinical data and gene expression profiles of patients with CRC from databases. The single-cell transcriptomic data was applied to identify M2-like TAM-related differentially expressed genes. Univariate Cox and least absolute shrinkage and selection operator regression analyses were used to determine the prognostic signature genes. Then, seven key genes were screened to develop the prognostic signature. In the training and external validation cohorts, the overall survival (OS) of patients in the high-risk group was significantly shorter compared to the low-risk group. Consequently, we created a nomogram that could accurately and reliably predict the prognosis of patient with CRC. A significant correlation was observed between the patient’s prognosis, clinical features, sensitivity to anticancer drugs, TME, and risk scores. Moreover, risk score was strongly related to the response to immunotherapy in patients from GSE91061, GSE78220, and GSE60331 cohorts. Finally, high expression of *HSPA1A*, *SERPINA1*, *CXCL1*, and low expression of *DNASE1L3* were found in human CRC tissue and normal tissue by using qRT-PCR. In conclusion, the M2-like TAM-related prognostic signature could predict the survival, prognosis, and response of patients with CRC to immunotherapy, which sheds light on the role of TAMs in CRCs and enhances our understanding of TAMs.

**Supplementary Information:**

The online version contains supplementary material available at 10.1186/s12876-023-03035-4.

## Introduction

Colorectal cancer (CRC) is the third most prevalent cancer worldwide [1]. It accounts for 10.2% of incidences and 9.2% of mortalities associated with cancers [2]. Despite advancements in therapeutic strategies for CRC, the prognosis of patients is still poor [3]. Furthermore, our understanding of immunotherapy in CRC is still limited. Therefore, it is necessary to explore and identify biomarkers that can predict the prognosis and aid in designing treatment strategies for patients with CRC.

Numerous studies have shown that tumor microenvironment (TME) contributes to tumor progression in CRC [4, 5]. In fact, most cells in TME are immune cells. Among all immune cells infiltrating TME, one of the most abundant immune cell types is tumor-associated macrophage (TAM) [6]. M2-type macrophages secrete high levels of anti-inflammatory stimuli and cytokines, like IL-10 and TGF-β1, that stimulate tumor growth and progression [5, 7]. TAMs often exhibit the M2 phenotype. Recent studies have shown an association between high macrophage levels and poor prognosis as well as an advanced stage of CRC. Badawi et al. demonstrated a high infiltration level of macrophages in TME of patients with colon carcinoma compared to adenomatous colon polyps [8]. Furthermore, M2 macrophage-derived exosomes transfer specific miRNAs to cancer cells, thus promoting invasion and metastasis of cancers like CRC [9, 10]. However, to date, no TAM-related biomarkers have been discovered and confirmed that can accurately predict the prognosis of patients suffering from CRC.

Recently, immunotherapy has been widely used as an optimal therapeutic approach for treating patients with carcinomas. M2-like TAMs display immunosuppressive characteristics [10]; hence the concurrent use of anti-M2 macrophages and immune checkpoint inhibitors (ICIs) could improve the therapeutic efficacy of existing strategies and may also aid in designing new therapeutic approaches [11]. Antitumor immunity is activated via ICIs, such as antibodies targeting cytotoxic T-Lymphocyte antigen-4 (CTLA-4) and anti-programmed cell death 1 (PD-1) [7, 12]. Hence, elucidating the role of TAMs in the pathogenesis of cancer will help predict the prognosis and develop new immunotherapeutic strategies for treating patients with CRC.

However, the mechanism underlying the impact of TAMs on TME characteristics and the efficacy of immunotherapy in CRC is still unexplored. In this study, we have analyzed the single-cell, and bulk RNA sequencing data of patients with CRC. Next, we identified a novel M2-like TAM-related prognostic signature and constructed a reliable nomogram to predict the OS of patients with CRC. Further, we explored the potential intercorrelations between clinical features, TME, response to immunotherapy, and risk scores. Our results showed a link between clinical outcomes and response to immunotherapy in patients with CRC and M2-like TAMs.

## Materials and methods

### Data acquisition and processing

The GSE132465 dataset consisting of single-cell RNA-sequencing (scRNA-seq) data of 33 samples (23 primary CRC and 10 normal tissues) were retrieved from the Gene Expression Omnibus (GEO) database. The processing of scRNA-seq data was performed using the “Seurat” package [13]. The cells expressing < 15% mitochondrial genes and with > 200 genes detected in > 3 cells were retained. Principal component analysis (PCA) and uniform manifold approximation and projection (UMAP) were used for identifying clusters. Different cell clusters were annotated using the “SingleR” package [14] and the CellMarker database. The bulk RNA sequencing and clinical data of patients with CRC (READ and COAD) were obtained from The Cancer Genome Atlas (TCGA) database. The datasets retrieved from GEO, like GSE39582, were used as independent validation cohorts, and GSE91061, GSE78220, and GSE60331 were used as immunotherapy cohorts.

### Identification and enrichment of differentially expressed genes (DEGs)

We screened M2-like TAM-related DEGs (M2RDEGs) from the M2 clusters between CRC and normal tissues using the “FindMarkers” function of the “Seurat” package. The “limma” package was used to screen M2RDEGs on the basis of the following parameters: P.adj < 0.05 and |Log2fold change| > 1. The volcano plot was generated using the “ggplot2” package. The “heatmap” package was used for creating the heatmap plot. The “clusterProfiler” package [15] was used for performing functional enrichment analyses.

### Construction and validation of the risk model

We conducted univariate Cox regression analysis to investigate the correlation between M2RDEGs and the OS of patients. The threshold of P value was 0.05. The “glmnet” package was used to perform “least absolute shrinkage and selection operator (LASSO) regression analysis” to determine the optimum signature genes and construct a risk model using expression profiles obtained from TCGA-CRC cohort. The following formula was applied to compute the risk score:

$${\varSigma }_{i=1}^{n}={exp}\text{i}\times \beta \text{i}$$.

Here, the *βi*: the regression coefficient for a gene.

*expi*: the expression value of a prognostic gene for a patient.

*n*: the number of prognostic genes.

The median risk score was utilized to split the patients with CRC into high- and low-risk groups. Kaplan-Meier (KM) survival analysis was performed for survival difference of cohorts with different risk score using the “Survival” and “survminer” packages. The “survivalROC” package was applied to create the Receiver operating characteristic (ROC) curves.

### Gene set variation analysis (GSVA)

The “GSVA” package was required to perform GSVA. MsigDB database was used to obtain reference gene sets like Gene Ontology (GO) gene sets biological processes (BP), GO cellular processes (CC), molecular processes (MP), and Kyoto Encyclopedia of Genes and Genomes (KEGG) gene sets [16]. The pathways with P.adj < 0.05 were deemed to be statistically significantly different.

### Correlation analysis between risk score and TME

We analyzed the expression of immune checkpoints in patients in two groups. Then, the “ESTIMATE” algorithm was utilized to obtain the stromal and immune scores for all patients. The “ssGSEA” package was used to calculate EMT scores based on the expression of epithelial-mesenchymal transition (EMT)-related genes. The stemness index per mRNA expression (mRNAsi) was obtained from the Progenitor Cell Biology Consortium database [17].

### Risk score predicts immunotherapy response and anticancer drug sensitivity

Patients in immunotherapy cohorts were classified into partial (PR) and complete responses (CR), progressive (PD) and stable diseases (SD) subgroups. According to the median risk score, these patients were separated into high- and low-risk groups. The half-maximal inhibitory concentrations (IC_50_) values of different chemotherapeutic drugs were calculated using the “pRRophetic” package [18].

### Relationship between risk scores and clinical features

For the clinical correlation analysis, the relationship between clinicopathological parameters like tumor and pathologic TNM stage and risk scores. The differences of risk score among three or more than three groups were analyzed using Kruskal–Wallis test and exhibited through “ggplot2” package. Wilcoxon-ranked sum test was employed to investigate the differences between two groups.

### Establishment and assessment of the nomogram

We integrated the risk scores and clinical features, and performed univariate and multivariate Cox regression analyses. The “regplot” package was used for constructing a nomogram. We created a calibration plot using the “rms” package.

### Collection of clinical samples

Five human CRC and adjacent normal tissues were obtained from patients with CRC and received surgery at The Affiliated Hospital of Youjiang Medical University for Nationalities from December 2022 to February 2023. The utilization of clinical samples followed the guidelines of the Ethics Committee of the hospital. Informed consent was obtained from all participants.

### Validating the expression of signature genes using quantitative real-time PCR (qRT-PCR)

Total RNA was extracted from clinical tissue samples (CRC and pared normal tissue) referenced to our previous study [19]. Then, qPCR was performed with an SYBR Green Master mix (Q111-02; Vazyme Biotech; Nanjing, China). The reactions repeated three times. Gene expression was quantitated via the 2 − ΔΔCt method and then normalized to GAPDH.

### Statistical analyses

R 4.1.3 and Rstudio 2022.02.0 were used to perform data and statistical analyses. Spearman’s coefficient was used to perform correlation analyses. Wilcoxon-ranked sum test was used to investigate the differences between two groups. The differences among three or more than three groups were analyzed using Kruskal–Wallis test. * P < 0.05, ** P < 0.01, *** P < 0.001, or **** P < 0.0001 was used to indicate statistical significance.

## Results

### Processing of scRNA-seq data

**Fig. 1:**
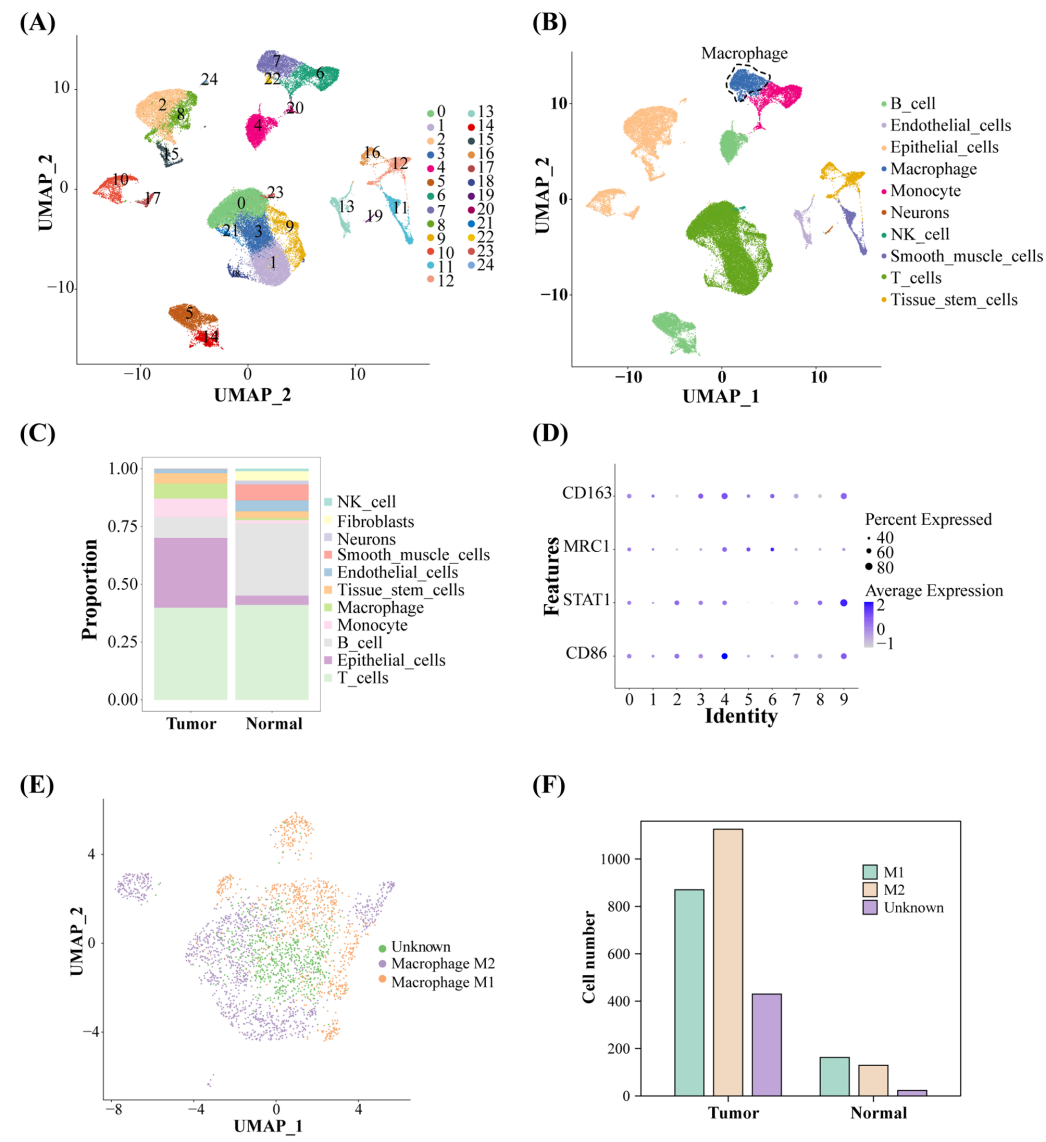
Processing of scRNA-seq data. **(A)** UMAP plot shows all the single cells from 33 CRC samples. **(B)** Annotation of cell clusters. M2 macrophages are shown in deep blue and circled by a dotted line. **(C)** A histogram shows the proportion of different cell types in tumor and normal subsets. **(D)** The expression of four marker genes in ten macrophage clusters. **(E)** UMAP plot of three cell sub-types of macrophages, M1 macrophage, M2 macrophage, and the unknown. **(F)** The cell number of different types of macrophages in tumor and normal subsets

After quality control, a total of 56,652 cells and 25,655 genes were used for downstream analysis. More detailed information of quality control is showed in Supplementary Fig. 1A-D. Twenty-five cell clusters were identified (Fig. 1A, Supplementary Fig. 2A). Overall, we identified 10 cell types, including B cells, macrophages, epithelial cells, endothelial cells, monocytes, neurons, NK cell, smooth muscle cells, tissue stem cells and T cells (Fig. 1B). Figure 1 C shows the proportion of different cell types in tumor and normal subsets. Macrophages were further divided into 10 clusters. *CD86* and *STAT1* were the specific markers for M1 macrophages, and *MRC1* and *CD163* were the markers for M2 macrophages [20, 21]. The expression of four marker genes in ten clusters is shown in Fig. 1D and Supplementary Fig. 2B. All macrophages were classified into three sub-types: M1, M2, and the unknown (Fig. 1E). The cell number of different types of macrophages is shown in Fig. 1F. The results revealed high infiltration of macrophages, specifically M2-like TAMs in tumor tissue, thus indicating the significance of M2 macrophages in tumor progression.

### Identification and functional enrichment of M2RDEGs

**Fig. 2:**
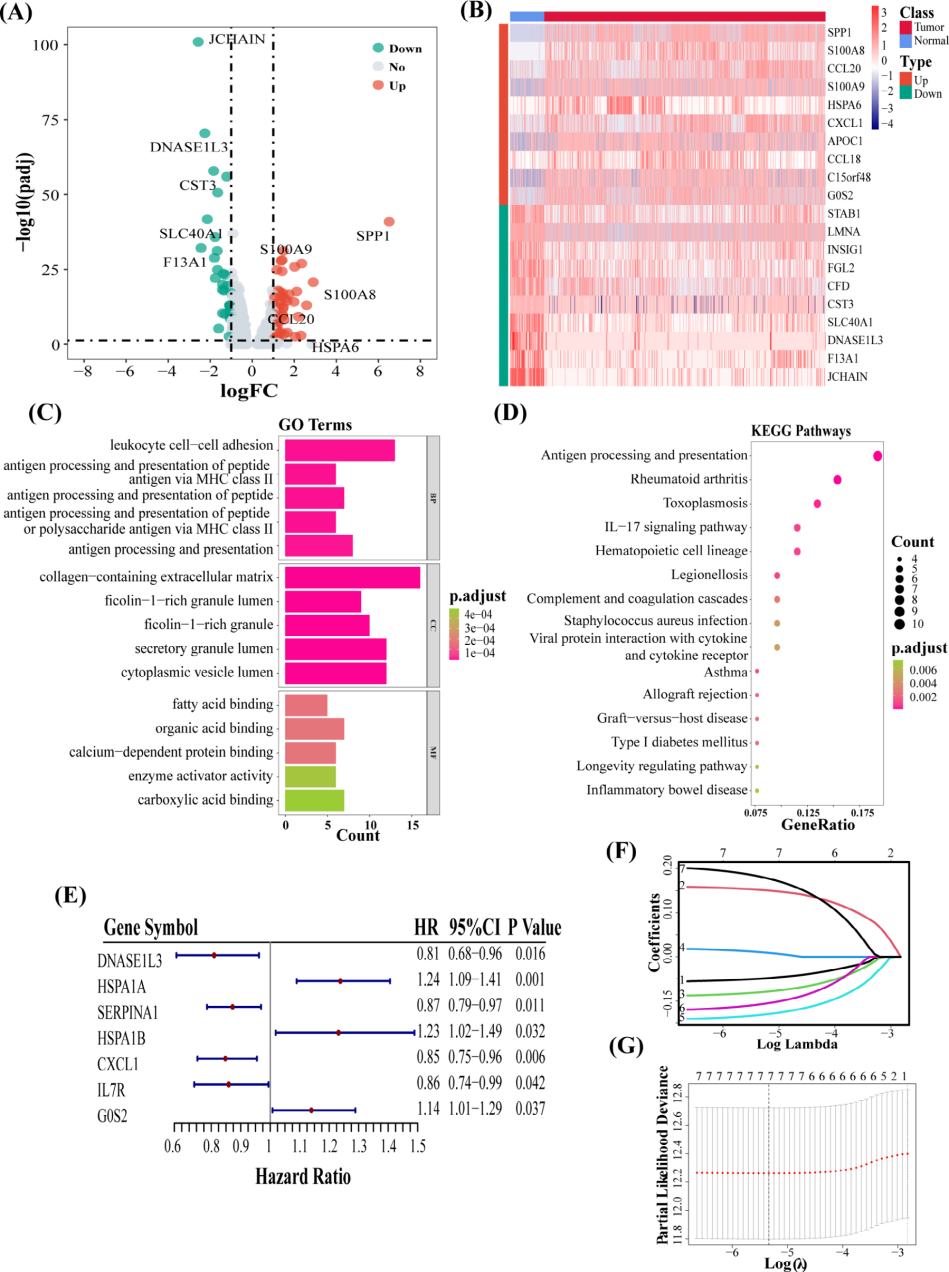
Identification and functional enrichment of M2RDEGs. **(A)** A volcano plot shows M2RDEGs that are upregulated (red) or downregulated (green) in M2 clusters between tumor and normal tissues. The top five upregulated genes and top five downregulated genes are labeled. **(B)** A heatmap shows the top 20 significantly expressed M2RDEGs. **(C)** GO and KEGG [16] **(D)** enrichment results of the 80 M2RDEGs. Identification of prognostic signature genes. **(E)** The forest plot shows the results of univariate cox regression of seven prognostic genes. Genes with Hazard Ratio < 1 were negatively associated with prognosis, and vice versa. **(F**, **G)** Lasso COX regression analysis was conducted to identify the best prognostic signature genes for constructing the risk model

We identified 80 M2RDEGs, of which 48 DEGs were upregulated, and 32 were downregulated (Fig. 2A). The heatmap shows the expression of the top 20 M2RDEGs (Fig. 2B). GO results indicated that M2RDEGs were enriched in processes like leukocyte cell-cell adhesion, antigen processing and presentation, and collagen-containing extracellular matrix (Fig. 2C). The KEGG results showed that M2RDEGs were enriched in the interleukin-17 signaling pathway and pathways linked to antigen processing and presentation (Fig. 2D). These results revealed that M2RDEGs were strongly correlated with biological processes and pathways associated with the immune system.

### Construction and validation of the risk model

Univariate Cox regression analysis revealed a significant correlation between seven M2RDEGs and the prognosis of patients (Fig. 2E). Next, LASSO regression analysis was performed on these seven M2RDEGs (Fig. 2F-G). The risk scores were calculated using the following formula:

risk score = (-0.050 * *DNASE1L3* exp) + (0.152 * *HSPA1A* exp) + (-0.080 * *SERPINA1* exp) + (0.011 * *HSPA1B* exp) + (-0.132 * *CXCL1* exp) + (-0.108 * *IL7R* exp) + (0.182 * *G0S2* exp).

**Fig. 3:**
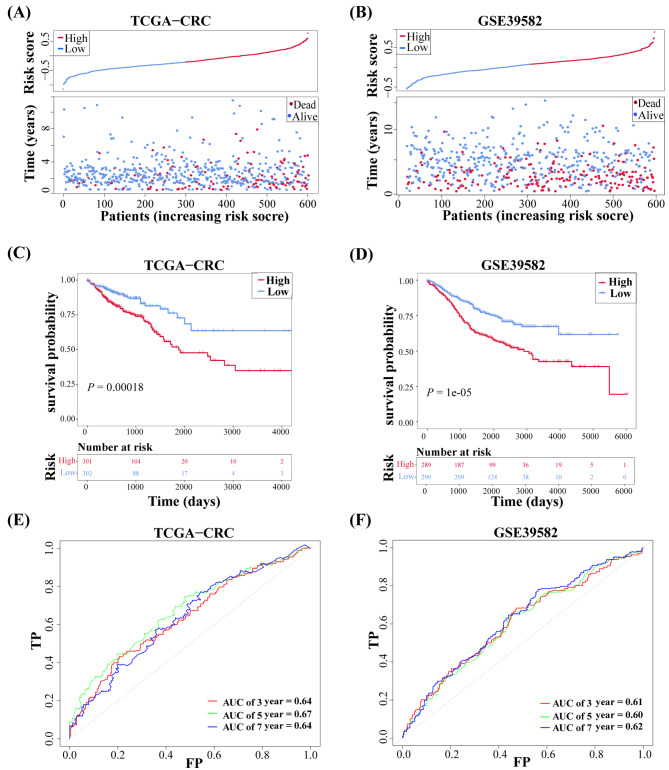
Validating the risk model. **(A, B)** Risk **score** distribution and scatter plots of the survival status in patients from TCGA-CRC and GSE39582 cohorts. Blue dots denote low risk scores, and red dots denote high risk scores. **(C, D)** Kaplan–Meier survival curves show a significant difference in the prognosis of patients from TCGA-CRC and GSE39582 cohorts. The prognosis of patients in the low-risk groups was better. **(E, F)** ROC curves and their AUC values for 3-, 5-, and 7-year OS.

The patients with high risk score in TCGA-CRC (Fig. 3A) and validation cohorts (Fig. 3B) had a shorter survival time. The KM survival curves show a significantly more unfavorable OS of patients in the high-risk compared to the low-risk group (Fig. 3C-D). The ROC curve indicates that the predictive efficacy of risk score was good. In TCGA-CRC cohort, the AUC values for the 3-year period were 0.64, 0.67 for the 5-year period, and 0.64 for the 7-year period (Fig. 3E). In the GSE39582 cohort, the AUC values for the 3-year were 0.61, the 5-year was 0.60, and the 7-year was 0.62 (Fig. 3F). Overall, the risk score demonstrated robust accuracy in predicting the prognosis of patients in both the training and validation cohorts.

### GSVA analysis between two risk groups

**Fig. 4:**
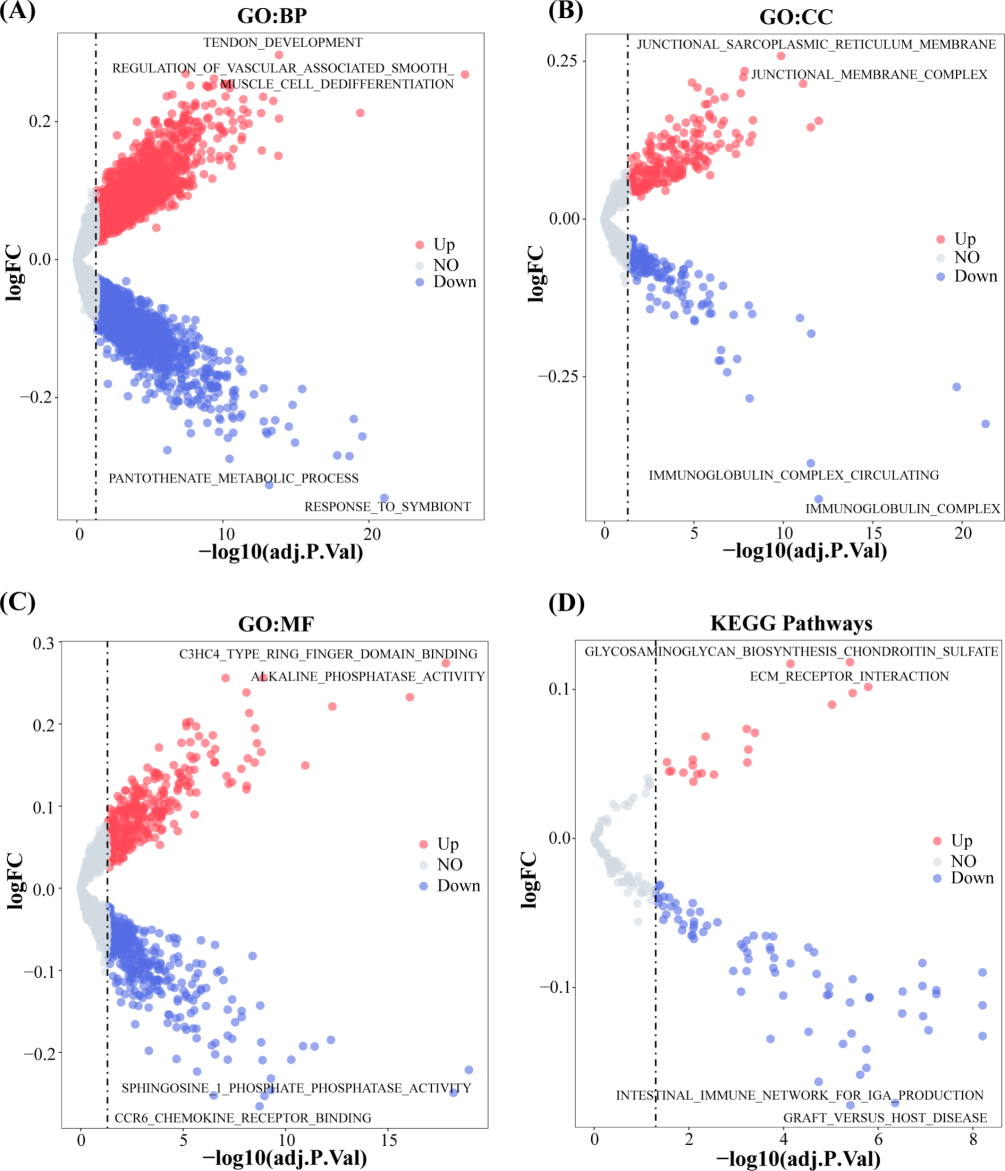
Gene set variation analysis (GSVA). **(A-D)** The results of GO: BP, GO: CC, GO: MF and KEGG pathway enrichment analysis

In patients in the low-risk group, the pathways like CCR6 chemokine-receptor binding were enriched, and the pathways like ECM receptor interaction were enriched in the high-risk group. These pathways are, to some extent, related to the biological process of immune activation; hence, their involvement should be investigated further. Figure 4 A-D shows the top two most significantly different pathways.

### Correlation between risk score and TME

**Fig. 5:**
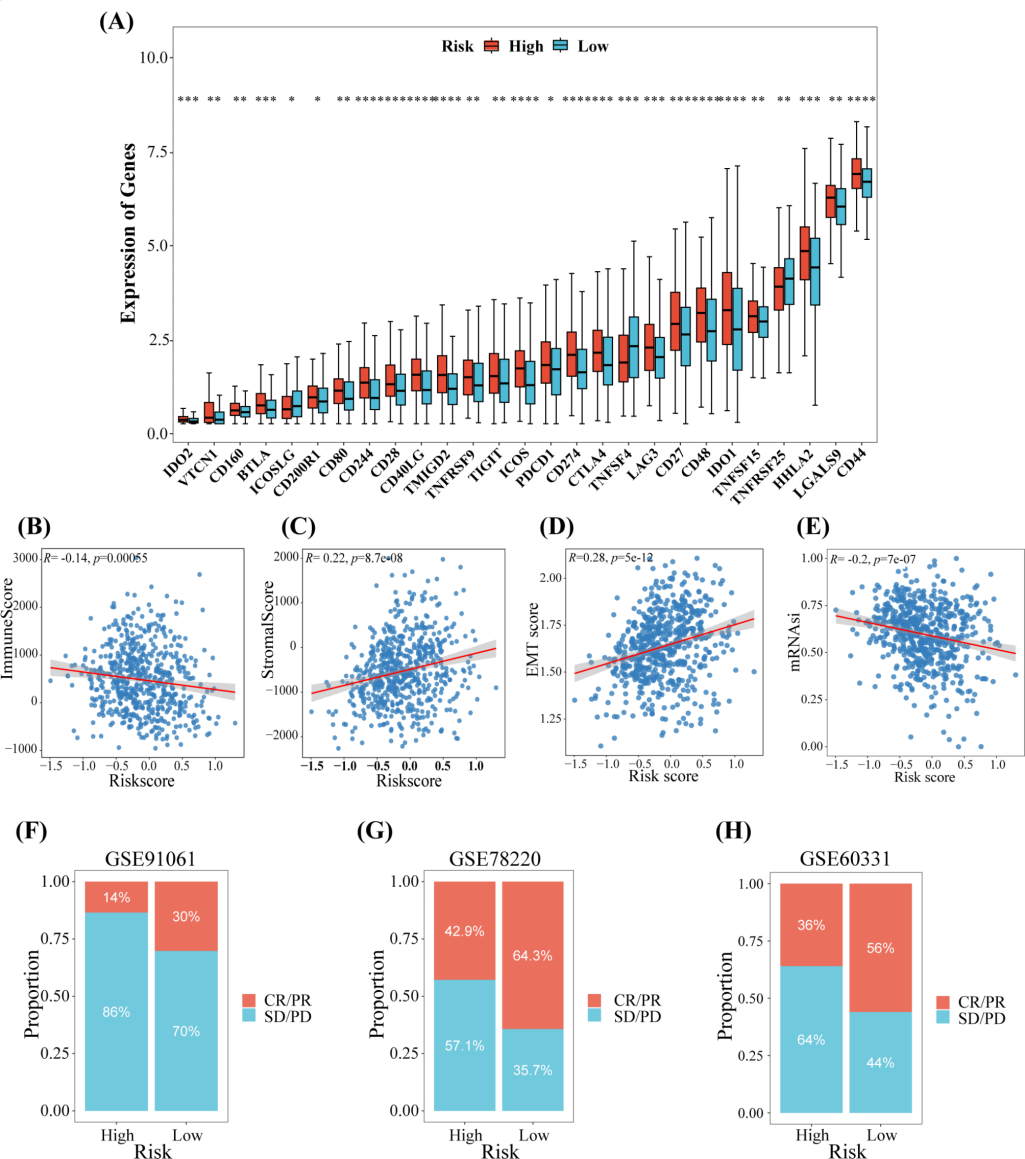
Correlation between tumor immune microenvironment, immunotherapy response and risk score. **(A)** The expression of immune checkpoint genes in patients in the two risk groups from TCGA-CRC. Spearman’s correlation analysis of the immune score **(B)**, stromal score **(C)**, EMT score **(D)**, mRNAsi **(E)** and risk score. **(F)** A bar plot shows the proportion of CR/PR and SD/PD in patients in the high- and low-risk groups from GSE91061, GSE78220 **(G)**, and GSE60331 cohort **(H)**. * *P* < 0.05, ** *P* < 0.01, *** *P* < 0.001, **** *P* < 0.0001

A total of 24 immune checkpoint genes expressed differently. The expression of *CTLA4, LGALS9*, and *CD44* were significantly higher in patients in high-risk group (Fig. 5A), whereas *TNFRSF25, TNFSF4*, and *ICOSLG* were highly expressing in low-risk group. Figure 5B demonstrated a significant (P = 0.0006, *R* = -0.14) negative correlation between the immune scores and risk scores. Figure 5 C shows a significant positive correlation (P = 8.7e-08, *R* = 0.22) between the stromal scores and risk scores. Further, a significant positive correlation (P = 1.6e-11, *R* = 0.27) was observed between the EMT scores and risk scores (Fig. 5D), whereas a significant (P = 7.1e-07, *R* = -0.2) negative correlation was observed between mRNAsi and risk scores (Fig. 5E). These results indicate a difference in the tumor immune microenvironment between high-risk and low-risk groups. Hence, novel targets for immunotherapy should be explored.

### Risk score predicts immunotherapy response and anticancer drug sensitivity

**Fig. 6:**
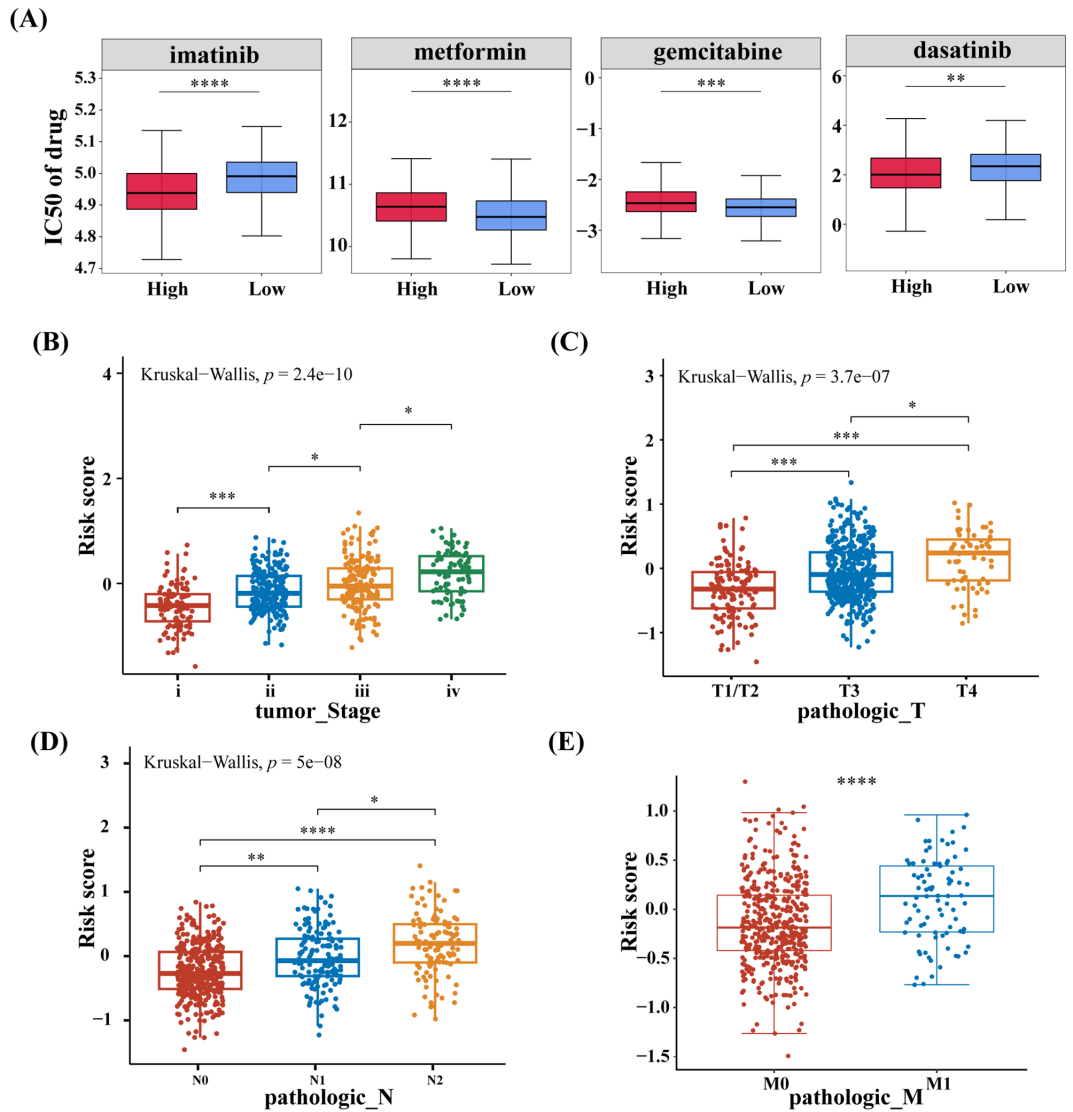
Drug sensitivity and clinical features. **(A)** The IC50 values of dasatinib, imatinib, gemcitabine and metformin in two risk groups. The relationship between risk score and clinicopathological characteristics: Tumor stage **(B)**, pathologic T **(C)**, pathologic N **(D)** and pathologic M **(E)**. (Wilcoxon and/or Kruskal-Wallis test). * *P* < 0.05, ** *P* < 0.01, *** *P* < 0.001, **** *P* < 0.0001, ns no statistical difference

We further analyzed if risk scores could predict a patient’s response to immunotherapy. Figure 5 F-H shows the number of patients who responded or did not respond to immunotherapy in three independent immunotherapy cohorts. A higher CR/PR proportion was observed in the low-risk group compared to the SD/PD group. The chemotherapeutic drugs with lower IC_50_ values had higher efficacy in treating cancer. Figure 6 A shows that patients in the high-risk group could be highly sensitive to dasatinib and imatinib. Furthermore, patients in the low-risk group could be more sensitive to gemcitabine and metformin. These results may help in designing therapeutic strategies on the basis of the risk score of patients.

### Relationships between risk score and clinical features

Figure 6B-E shows a significant positive link between the risk score and tumor stages (I, II, III, and IV), pathologic T stages (T1/T2, T3, and T4), pathologic N stages (N0, N1, and N2) and pathologic M stages (M0, M2). Significant correlations were observed between risk score and clinical parameters. In addition, stratified analyses were performed to analyze the survival differences between patients with different clinical characteristics in different risk groups. In the survival analysis based on pathologic T stage, there was a significant difference between the high- and low-risk groups at T3. According to pathologic M stage, there was a significant difference at M0 stage (Supplementary Fig. 3). Thus, a high risk score could indicate tumor progression and poor clinical outcomes.

### Establishment and assessment of the nomogram

**Fig. 7:**
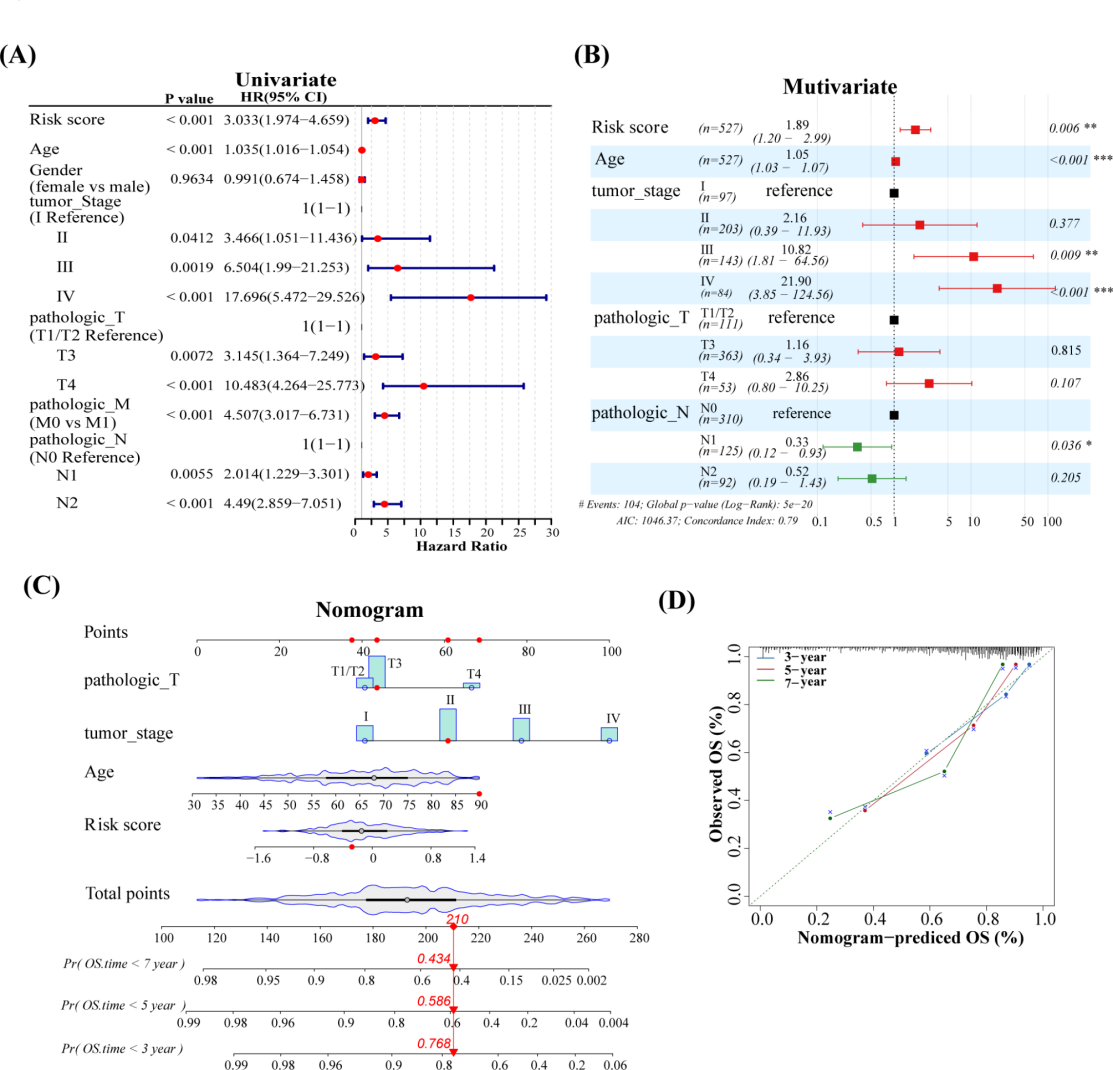
Construction and assessment of nomogram. **(A, B)** Univariate and multivariate Cox regression analyses of prognostic factors in TCGA cohort. **(C)** Nomogram was constructed by incorporating risk score and clinical indicators. **(D)** The calibration curve showed that the nomogram had good accuracy in predicting prognosis

Univariate COX regression and multivariate COX regression analyses demonstrated a significant correlation between the risk scores, age, tumor stage, pathologic TNM stages, and the survival of patients with CRC (Fig. 7A-B). Next, a nomogram was constructed, which could better predict the patient’s prognosis (Fig. 7C). The calibration curve indicated that the nomogram demonstrated great accuracy and was reliable in predicting the prognosis of patients with CRC (Fig. 7D).

### Expression of signature genes and validation by qRT-PCR

**Fig. 8:**
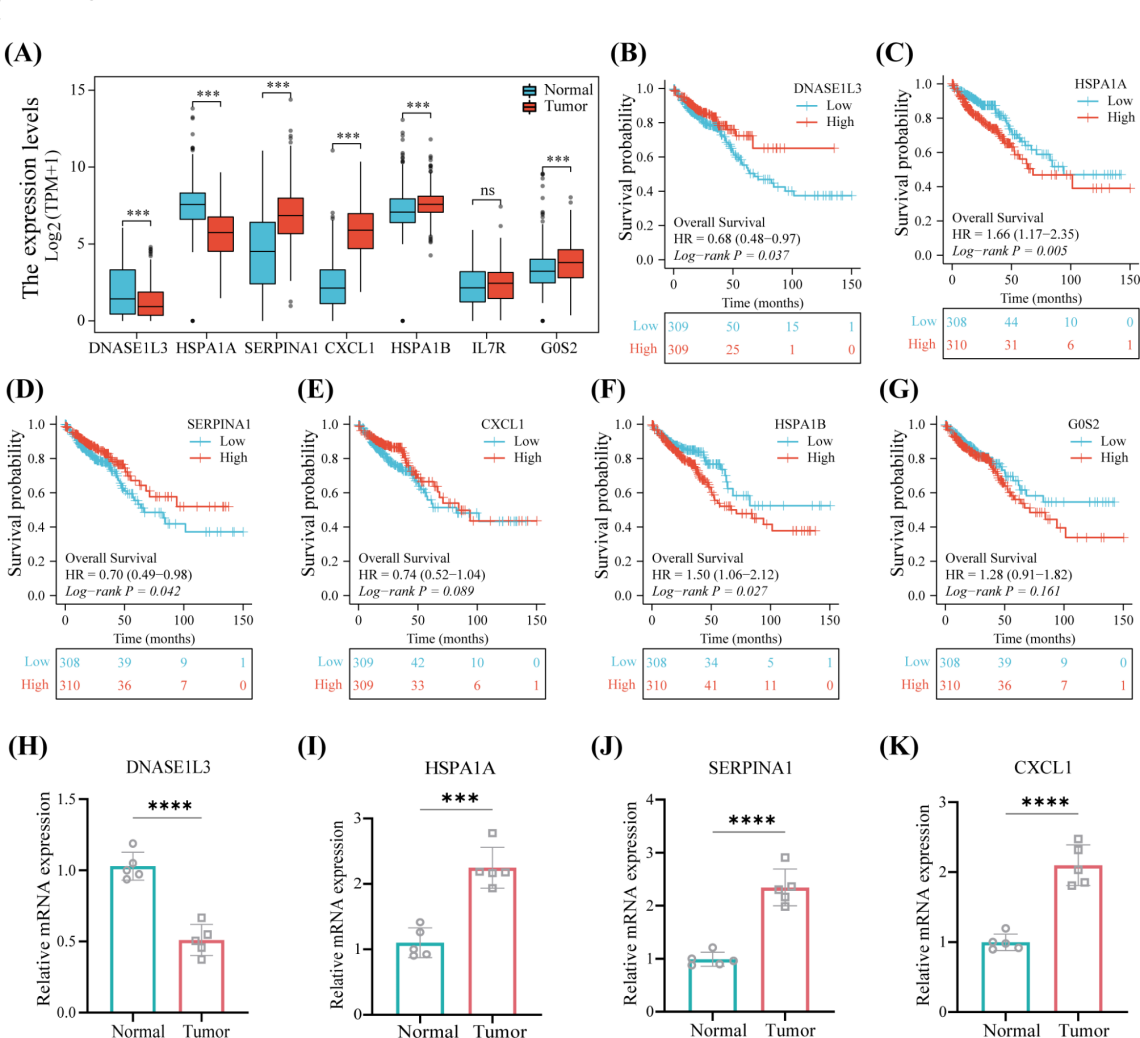
Investigating the signature genes and validation using qRT-PCR. **(A)** The mRNA expression of DNASE1L3, HSPA1A, SERPINA1, HSPA1B, CXCL1, IL7R, and G0S2 in patients from TCGA-CRC. **(B-G)** Kaplan–Meier survival curves of seven signature genes showed significant difference of prognosis in TCGA. **(H-K)** The mRNA expression of DNASE1L3, HSPA1A, SERPINA1, and CXCL1 were analyzed by qRT-PCR with human CRC and normal tissue. * *P* < 0.05, ** *P* < 0.01, *** *P* < 0.001, ns no statistical difference

The mRNA expression of seven prognostic signature genes in patients in the TCGA-CRC were analyzed (Fig. 8A). All genes, excluding *IL7R*, shows significant expression differences. Further, K-M curves were plotted to investigate the correlation of signature genes expression and patient survival based on the corresponding overall survival (OS), disease-specific survival (DSS), disease-free interval (DFI), and progression-free interval (PFI) data. As showed in the KM plots (Fig. 8B-G), high expression of *DNASE1L3* and *SERPINA1* indicates a better prognosis of patients with CRC. Whereas patients with high expression of *HSPA1A* and *HSPA1B* had a low probability to survive. Likewise, in DSS analysis, except for *DNASE1L3* and *IL7R*, the significant survival differences of other genes were observed based on high- and low-expression groups. In DFI analysis, except for *G0S2*, the survival difference of other genes was significant. In PFS analysis, all genes showed significant differences between high- and low-expression groups (Supplementary Figs. 4–6).

Then, qRT-PCR analysis was performed. The mRNA expression of *DNASE1L3* is lower in human CRC tissue than that in normal tissue (Fig. 8H). On the contrary, *HSPA1A*, *SERPINA1* and *CXCL1* are highly expressed in human CRC tissue (Fig. 8I-K).

## Discussion

The TME plays a crucial role in the onset progression of various cancers [22, 23]. Macrophages are a major immune cell type of TME and are involved in angiogenesis, invasion, and metastasis of cancer cells, regulation of the TME, and drug resistance [24]. Mounting evidence has suggested the involvement of TAMs in the interactions between TME and CRC cells [25–27]. Nevertheless, few studies have explored multiple TAM-related prognostic markers and the relationship between TAMs and TME in CRC.

In current study, we investigated the M2-like TAM-related genes combining single cell and bulk RNA-seq data of patients with CRC. We conducted univariate and LASSO Cox regression analyses to identify key genes affecting the progression of CRC. Seven prognostic signature genes were identified. Next, a risk model based on these seven prognostic signature genes was validated on independent validation cohorts and demonstrated good performance. A nomogram based on clinical features (age, tumor stage, pathologic T stage) and the risk scores could effectively predict patient prognosis. Finally, our results validated that the risk scores could be an independent predictor of the prognosis in patients with CRC. Higher mortality and higher/advanced tumor stage and pathologic T stage were observed in patients with higher risk scores. Together, this indicates that we developed a robust M2-like TAM-related prognostic signature that demonstrated good performance in predicting the prognosis and survival of patients with CRC, thereby contributing to developing M2-like TAM-related biomarkers.

Studies have shown the involvement of prognostic signature genes in tumor progression and metastasis, thereby contributing significantly to CRC onset and progression. For example, Serpin Family A Member 1 (*SERPINA1*) encodes for alpha-1 antitrypsin (A1AT) protein, which regulates the invasive and metastatic capacities of various cancers like lung, gastric, and CRC [28]. A study has shown a positive correlation between A1AT levels and the stage of CRC progression [29]. An increase of A1AT was observed in the blood of patients with CRC and has a superior accuracy and specificity than carcinoembryonic antigen [30]. Chemokine ligand 1 (*CXCL1*) is overexpressed in CRC. Zhou et al. performed immunohistochemistry and revealed a significant increase in *CXCL1* expression of CRC tissues compared to the adjacent normal tissues to determine *CXCL1* expression in CRC and normal tissues [31]. Additionally, *CXCL1* is an independent biomarker that could predict the prognosis of patients with CRC [2, 31]. Deoxyribonuclease 1-like 3 (*DNASE1L3*) is secreted by macrophages and could be an independent prognostic marker to predict survival outcomes in patients following radical liver cancer resection [32, 33]. In our study, the mRNA expression level of *DNASE1L3* is lower in the cancer samples than that in normal samples. On the contrary, *HSPA1A*, *SERPINA1* and *CXCL1* are highly expressed in tumor tissues. However, few studies have investigated the underlying mechanism of *HSPA1A, HSPA1B, IL7R*, and *G0S2* in CRC.

Immune cells form the primary component of the TME, of which macrophages account for approximately 30–50% of the total immune cells [9]. In our study, the levels of M2 macrophages were higher in CRC tissues compared to normal tissues. Previous studies have demonstrated that an increase in levels of M1 macrophages could be a protective factor, whereas high levels of M2 macrophages could be a risk factor for patients with CRC [27]. The results of our study are in line with previous studies. TAMs induce immune suppression by expressing inhibitory receptors ligands of immune checkpoints [27, 34]. An increase in the expression of *CTLA4* and Indoleamine-2,3-Dioxygenase (*IDO)* was observed in the patients in the high-risk group. Whereas in patients in the low-risk group, an increase in *TNFRSF25, TNFSF4*, and *ICOSLG* expression was observed. ICIs are promising treatment strategies for cancer and have been successfully exploited for treating various cancers such as bladder, lung, melanomas, and CRC [35]. Inhibiting *PD-L1, CTLA4*, and *LAG3* could increase the levels of CD8^+^ T cells and CD4^+^ T cells and decrease levels of regulatory T cells, thereby enhancing anticancer immune response [36]. High *IDO* expression level is observed in macrophages and tumor cells, and *IDO* expression indicates poor prognosis in patients [36, 37]. Currently, clinical trials are evaluating the efficacy of small-molecule inhibitors targeting *IDO* for reestablishing positive immune responses. During preclinical studies, a synergistic effect of ICIs and TAM repolarization was observed, which enhances the activation and infiltration of CD8^+^ cytotoxic T cells in tumors [38, 39]. Moreover, in our study, the patients in the low-risk group of independent cohorts showed a greater likelihood of responding to treatment with immunotherapy like ICIs, which confirms that the risk score could predict a patient’s response to immunotherapy. Growing evidence has shown a strong association between macrophages and immune, EMT, and stromal scores [6]. TAMs promote EMT and invasion of tumor cells by increasing cytokines and TGF-β levels. Furthermore, aberrant EMT activation is associated with tumor aggressiveness, cancer progression, and increased CRC relapse [6]. In our study, the risk score was distinctly related to EMT and stromal scores. TAMs induce chemoresistance by promoting cell survival and antiapoptotic signals, thereby inducing pro-tumor polarization in the TME [40]. A study has shown that Macrophages derived exosomes enriched in miR-223 induce chemoresistance in gastric tumor cells [41]. Thus, repolarization or ablation of M2-like TAMs could benefit cancer therapy. Our results demonstrated high sensitivity to dasatinib and imatinib in patients in the high-risk group, whereas patients in the low-risk group exhibited a higher sensitivity to drugs like gemcitabine and metformin. Interestingly, metformin is traditionally used to treat type П diabetes. Recently, studies have demonstrated that metformin mediates anti-inflammatory and anticancer effects. Kang et al. showed that metformin activates AMPK, downregulating the mevalonate pathway, thereby reducing the infiltration of M2 macrophage [5]. Thus, metformin could regulate immune cells in the TME; therefore, it could be considered as a promising prevention and treatment strategy for treating CRC. Together, these previous findings and our results indicates that treatment strategies using macrophages alone or in combination could improve their therapeutic efficacy.

However, our study has some limitations. First, tumor heterogeneity could cause sampling bias, leading to differences in macrophage levels in each sample. Secondly, there is more or less subjectivity while annotating the cell clusters. This is a common concern during single-cell analysis. Third, the underlying mechanism of TAMs and CRC cell interaction is yet to be explored; our results should be verified by performing multicenter clinical and further experimental studies. Finally, large sample size population-based prospective studies are required to enhance our understanding of TAMs and TAM-related prognostic signature.

In summary, we analyzed single-cell and bulk RNA sequencing data of patients with CRC to explore the promising M2-like TAM-related prognostic biomarkers for CRC. The new M2-like TAM-related prognostic signature could improve our understanding of the TME of CRC and could be used in clinical settings for predicting the prognosis and designing potential immunotherapeutic strategies for patients with CRC.

### Electronic supplementary material

Below is the link to the electronic supplementary material.

Supplementary Material 1

Supplementary Material 2

Supplementary Material 3

Supplementary Material 4

Supplementary Material 5

Supplementary Material 6

## Data Availability

The datasets analyzed during the bioinformatics analysis are available in the [GEO] database [http://www.ncbi.nlm.nih.gov/geo, accession numbers: GSE132465, GSE39582, GSE91061, GSE78220, and GSE60331], and the [TCGA] database [https://genome-cancer.ucsc.edu/]. The codes generated in this study are available from the corresponding author upon reasonable request.
