# Supplementary figures and images for: Integration of single-cell and bulk RNA sequencing to establish a prognostic signature based on tumor-associated macrophages in colorectal cancer
Source: BMC Gastroenterol. 2023 Nov 10;23:385. doi: 10.1186/s12876-023-03035-4 (PMC10638776; doi:10.1186/s12876-023-03035-4)

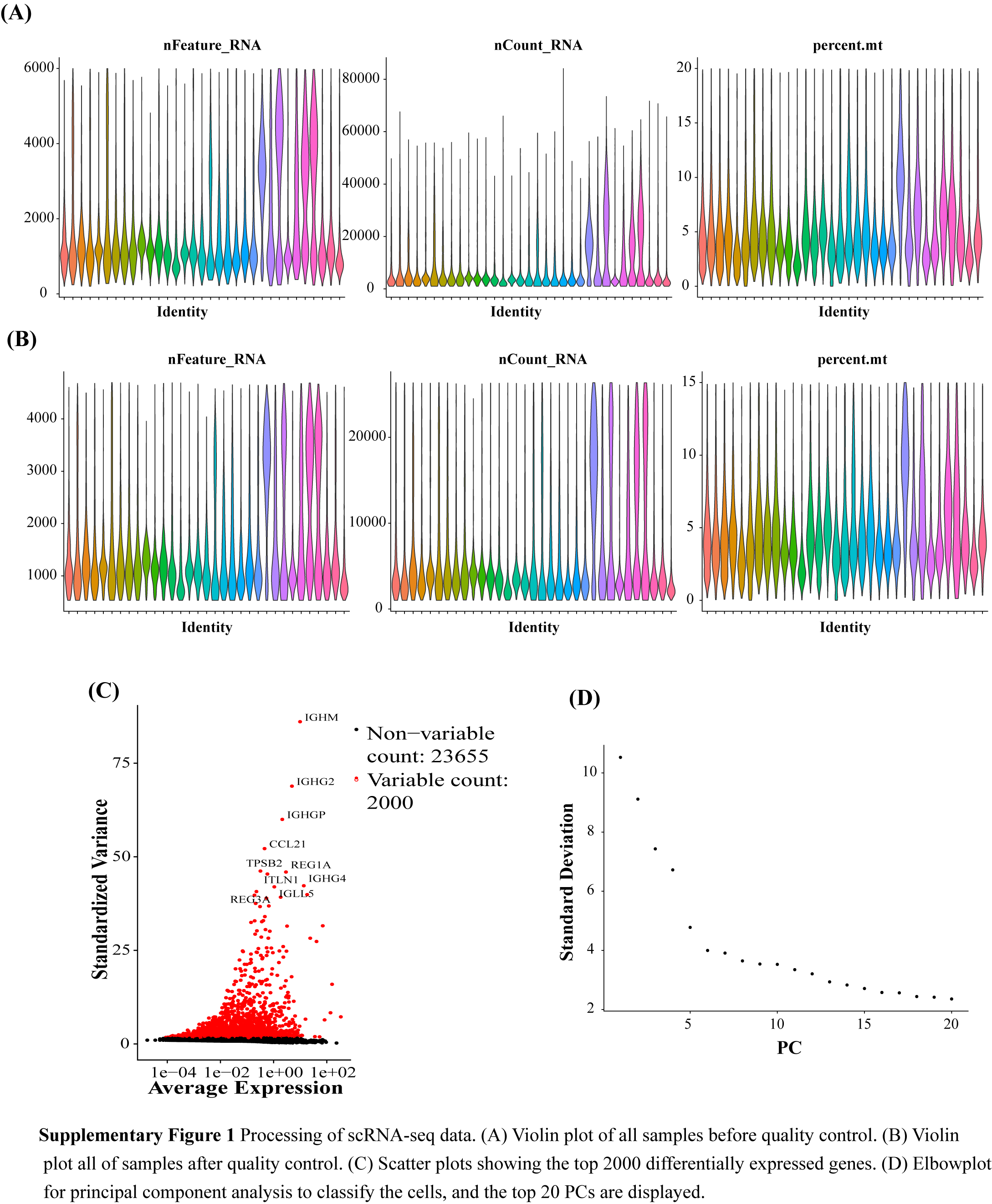

Supplement: Supplementary file 1 — Supplementary Material 1 [file 12876_2023_3035_MOESM1_ESM.png]

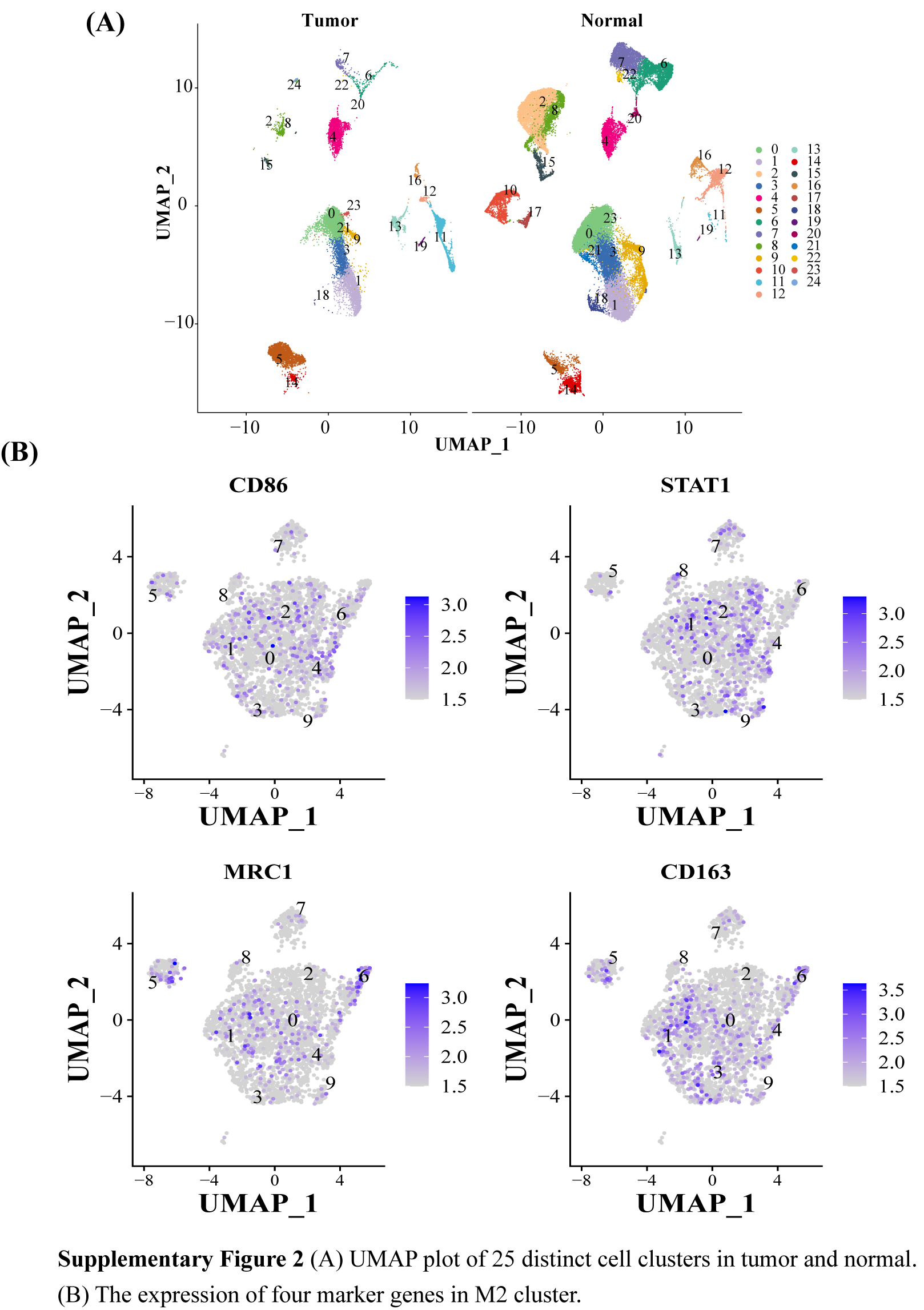

Supplement: Supplementary file 2 — Supplementary Material 2 [file 12876_2023_3035_MOESM2_ESM.png]

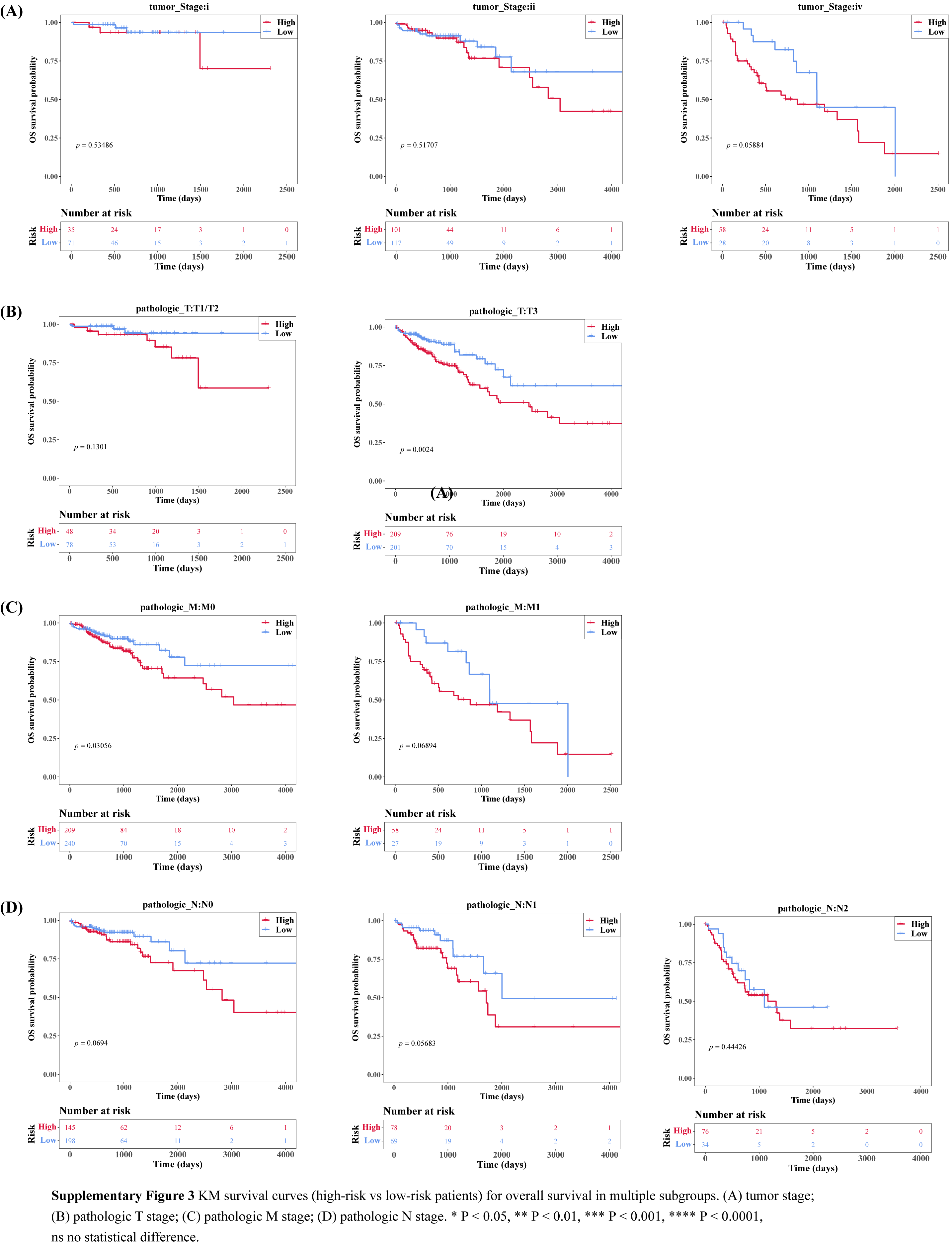

Supplement: Supplementary file 3 — Supplementary Material 3 [file 12876_2023_3035_MOESM3_ESM.png]

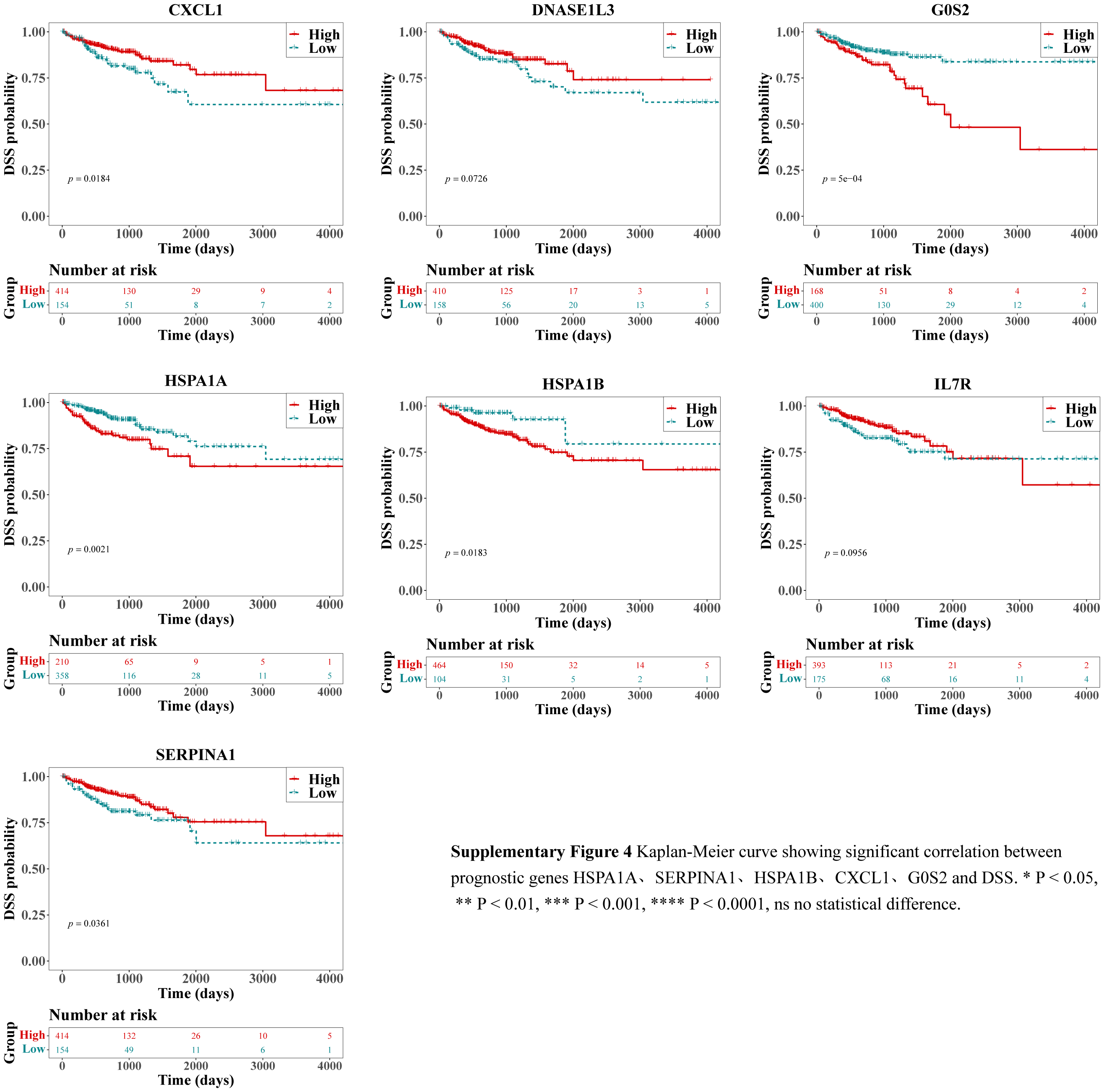

Supplement: Supplementary file 4 — Supplementary Material 4 [file 12876_2023_3035_MOESM4_ESM.png]

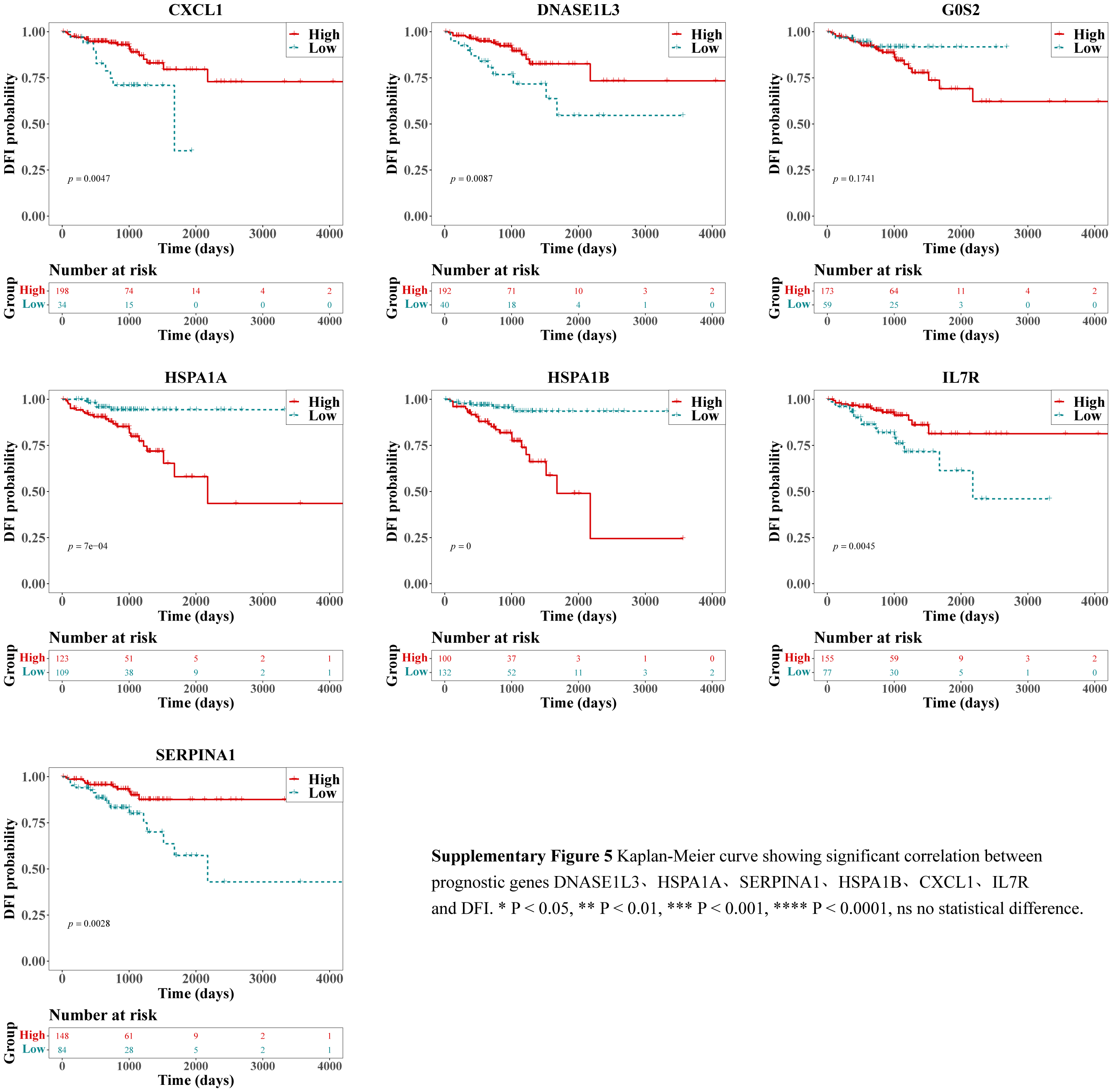

Supplement: Supplementary file 5 — Supplementary Material 5 [file 12876_2023_3035_MOESM5_ESM.png]

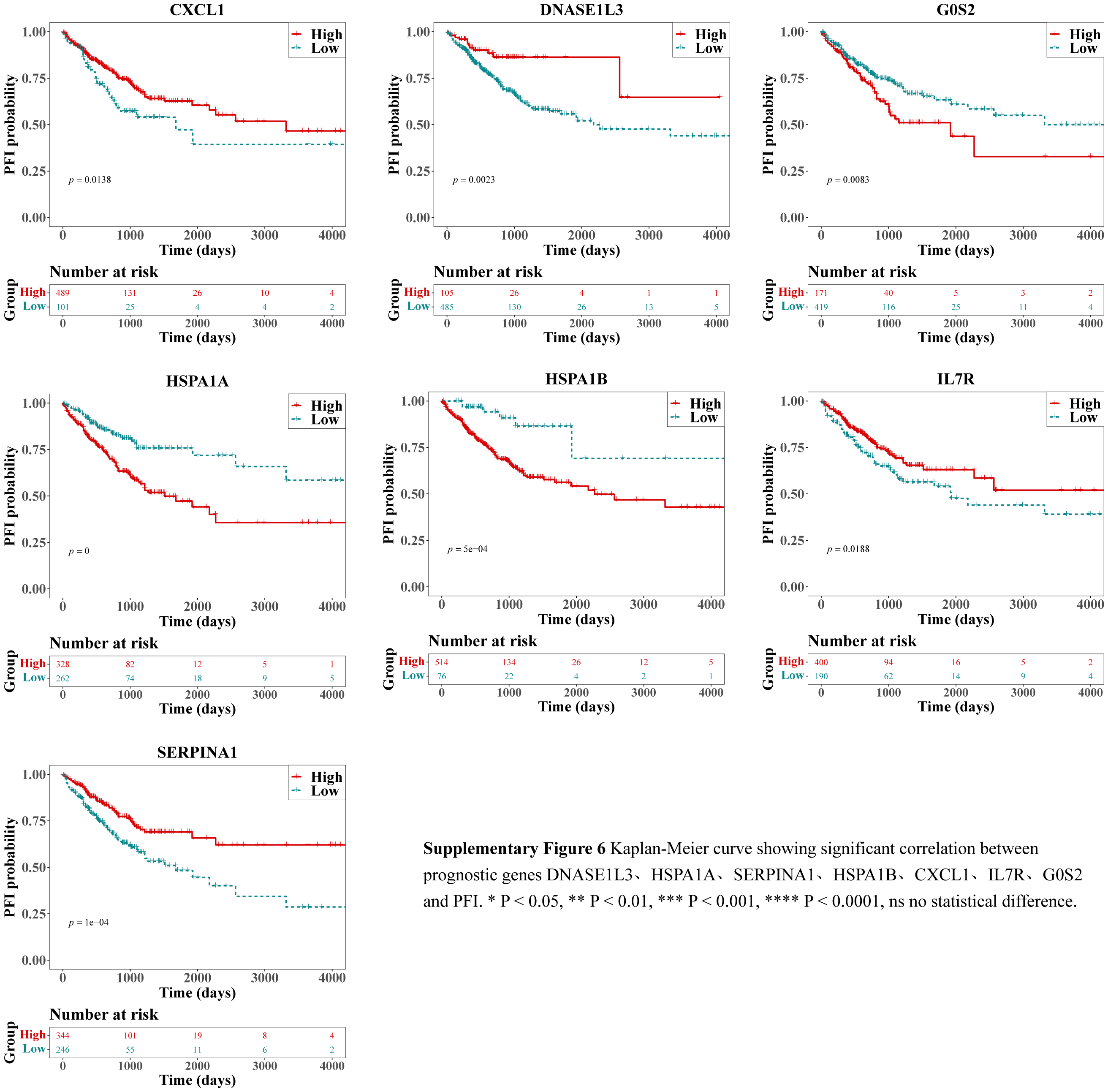

Supplement: Supplementary file 6 — Supplementary Material 6 [file 12876_2023_3035_MOESM6_ESM.png]
